# Dynamics of Lifestyle Counseling for Chronic Diseases Within and Between General Practices and Social Work Services *Causal Loop Diagram and Points for Improvement*

**DOI:** 10.1177/21501319251412648

**Published:** 2026-01-16

**Authors:** Demi E. van Os, Bart H. L. Ament, Suzanne A. Ligthart, Gerdine A. J. Fransen, Willem J. J. Assendelft

**Affiliations:** 1 Radboud University Medical Center, Nijmegen, The Netherlands; 2 AMPHI Academic Collaborative Centre, Nijmegen, The Netherlands

**Keywords:** primary care, community health, lifestyle change, health promotion, prevention, social determinants of health, disease management

## Abstract

**Background:**

A healthy lifestyle can slow the progression of chronic conditions and improve quality of life. Lifestyle counseling in Dutch general practices can be further optimized, among other factors by improving collaboration with social work services. To achieve this, it is important to map out the system of lifestyle counseling within and between general practices and social work services, as well as to identify opportunities for improvement.

**Methods:**

Semi-structured individual interviews were conducted with 3 general practitioners (GPs) and 6 practice nurses (PNs), as well as 5 group interviews with in total 15 professionals from social work services. Participants were based in the city of Nijmegen, the Netherlands. The interviews were conducted between March and August 2024. A thematic analysis was performed which resulted in themes and subthemes. Hereafter, the interviews were re-read to examine relationships between subthemes. The themes, subthemes, and relationships formed the basis for developing a causal loop diagram (CLD) and identifying areas for improvement. The CLD was subsequently reviewed through a member check with the same professions.

**Results:**

The system of lifestyle counseling within and between general practices and social work services consists of the following main themes; addressing lifestyle within general practices, referral to social work services from general practices, GP/PNs’ overview and knowledge about social work services, contact between GP/PNs and social work services, and patient status exchange between GP/PNs and social work services. These main themes include interrelated variables which facilitate or impede referrals from general practices to social work services, which is illustrated by the CLD. For example, the more patient status information is shared, the better the GP/PNs’ understanding of the available social work services.

**Conclusion:**

The CLD illustrates multiple factors that influence the system of lifestyle counseling in and between general practices and social work services. The CLD, together with the improvement points identified in the interviews, leads to actionable strategies to enhance collaboration between general practices and social work services. These strategies include increasing GP/PNs’ understanding of the role and activities of social work services, exchange information, as well as strengthening mutual familiarity and facilitating personal contact between professionals in both domains.

## Introduction

The global prevalence of type 2 diabetes mellitus (T2DM), cardiovascular disease (CVD), and chronic obstructive pulmonary disease (COPD) is rising.^1^ A healthy lifestyle not only helps to prevent these chronic diseases, but also reduces their progression and enhances overall quality of life of affected individuals.^2,3^ Preventing and managing chronic diseases is crucial for ensuring the sustainability of healthcare systems, which are currently facing strain.^1,4^ In many countries general practitioners (GPs) and practice nurses (PNs) play a crucial role in addressing the importance of lifestyle, serving as the primary and most accessible patient contact within the healthcare system. In addition, GPs and PNs are well positioned to provide longitudinal lifestyle counseling, which is important for achieving effective lifestyle change.^5^ In the Netherlands, patients with T2DM, CVD, and COPD are offered integrated care through structured care programs. These programs involve collaboration among multiple healthcare providers, such as GPs, PNs, dietitians, and physiotherapists. Within this integrated care framework, lifestyle counseling is an essential component.^6^ Lifestyle counseling includes aspects such as diet, physical activity, alcohol consumption, smoking, stress, sleep, and social connection, many of which are interrelated.^7-10^ GPs and PNs can provide lifestyle counseling themselves, or refer patients to other primary care services (dietitians and physiotherapists), social work services (providing non-medical support to improve well-being; eg, community sports coaches and community workers), and combined lifestyle interventions (CLI, reimbursed programs designed to support overweight individuals).^11-13^

Lifestyle counseling in general practices can be impeded by several barriers. Barriers in lifestyle counseling may stem from practical limitations as well as from the attitude of patients, GPs, and PNs. Most frequently cited barriers in studies conducted across European countries are patients’ lack of motivation, GPs’ and PNs’ time limitations, and inadequate knowledge and skills to effectively discuss lifestyle.^14-18^ Furthermore, general practices can make more use of the referral options to social work services. Social work services can help patients in addressing lifestyle issues arising from social determinants that are often beyond the scope of the medical domain, such as loneliness or housing problems.^19,20^ These kind of lifestyle issues can often be more appropriately addressed within social work services. To help connect general practices with social work services, there is a Dutch social prescribing program.^21^ Through this program, GPs and PNs can refer patients with psychosocial issues to wellbeing coaches who are closely connected to social work services. However, this program is solely targeted at wellbeing and not optimally integrated in care programs.

When aiming for improvement of lifestyle among patients with chronic diseases, strengthening the collaboration between general practices and these services is crucial.^19,22,23^ For GPs and PNs it is relatively uncommon to collaborate with the full range of social work services.^19,20,22^ Some of the barriers that hinder effective collaboration are described in literature, such as the fact that GPs and PNs often do not have a comprehensive understanding of social work services and how to access them.^22,24^ Additionally, there is a deficiency in mutual trust and a collaborative mindset among professionals in both domains.^19,20,22^ Also, collaboration between professionals needs to be supported more at both policy and organizational levels.^19,22^ To improve cross-domain collaboration between general practices and social work services it is crucial to gain a clear understanding of the current state of collaboration and its dynamics.

How general practices collaborate with social work services is influenced by various factors within the lifestyle counseling system. While previous studies have explored improvements for lifestyle counseling in general practices and also for the collaboration between general practices and social work services, none have provided an overview of the system and its complex interactions.^14,15,17-20,22,24^ The factors of the lifestyle counseling system (eg, having an overview or having trust in social work services) are likely to be interdependent. Therefore, a systems science approach, such as developing a causal loop diagram (CLD), helps to visualize and analyze the connections between the various factors influencing the lifestyle counseling system. A CLD is a visual representation that depicts the connections between factors through positive or negative links, illustrating how a change in 1 factor can reinforce or counteract changes in another.^25,26^ In this way, a CLD can provide insights into the current state of collaboration and indicate what improvements or changes could be implemented.

Consequently, the objective of this study is to map the current lifestyle counseling system and identify areas for improvement for patients with T2DM, CVD, and COPD within and between Dutch general practices and social work services. The aim is to do this from the perspective of general practices, since for a patient with a lifestyle-related disease this is the most likely starting point for lifestyle change. The Dutch context is relevant because of its integrated care programs, social prescribing program, and the relatively strong presence of social work services.

## Methods

### Design and Setting

This study follows a qualitative systems approach, using interview data to explore how professionals from general practice and social work services perceive and experience providing lifestyle counseling. Semi-structured interviews were conducted with general practitioners (GPs), practice nurses (PNs), and professionals from social work services to gather input for the development of a CLD and to identify points for improvement. To ensure its accuracy and relevance, the CLD was validated through a member-check process. This research focused on 3 districts in the city of Nijmegen, the Netherlands, which were selected to ensure socioeconomic diversity. This study is reported in accordance with the Standards for Reporting Qualitative Research (SRQR).^27^

### Participants

General practice owners and managers of social work services within the districts of focus were asked which professionals could be contacted for participation. For the general practices, all PNs were interviewed, while the practice owners selected which GPs could be interviewed. For 3 social work organizations, all available professionals were included, as each organization employs 1 professional per district. For the remaining 2 organizations, the managers determined which professionals could be interviewed. The interviewees were recruited through phone calls or email correspondence.

Semi-structured in-depth individual interviews were conducted with 3 GPs and 6 PNs, the latter specifically involved in somatic care for chronic diseases. Both professions were represented from the 3 specific districts.

Due to the wide range of roles within social services, 5 group interviews were held with a total of 15 professionals working within social services organizations. See Table 1 for more details on the professionals who participated in each interview. All of these professions operate at the district level. In the first 3 group interviews the 3 districts of focus were represented by at least 1 person. In both the fourth and fifth group interview 2 districts of focus were represented.

**Table 1.** Overview of Group Interview Participants and Their Main Tasks.

Group interview	Professions included	Main tasks of professions
1	Three community sports coaches	Supporting physical activity at both individual and community levels
2	Four community social workers from neighborhood teams	Providing wellbeing support at the individual levels
3	Two coordinators of community support services whereof one also served as a wellbeing coach within the social prescribing program	Coordinating community support services and for the wellbeing coach supporting wellbeing through conversations and referring individuals to local activities.
	Two community workers	Supporting Community development
4	Two health policy advisors from the Municipal Health Services	Advising on local public health and prevention
5	Two advisors elderly care	Supporting the well-being of elderly, mainly at the individual level

### Interview Procedure

The semi-structured individual interviews and the group interviews followed an interview guide. Interviewees were informed beforehand that the questions focused on lifestyle counseling for T2DM, CVD, and COPD patients. The interview guides were partially based on previous studies that focused on lifestyle advice and referrals within general practice settings.^11,14,15,28-30^ See Table 2 for an overview of the themes and probing questions included in the interview guide for individual interviews with GPs and PNs. The group interviews with workers from social services followed a slightly different interview guide, focusing primarily on the collaboration with general practices and other social work services, see Supplemental Appendix A.

**Table 2.** Topics and Examples of Probing Questions Outlined in the Interview Guide for Individual Interviews With General Practices.

Topics regarding lifestyle counseling	Examples of probing questions
Role	*• What is your role regarding lifestyle counseling? • What do you think about conducting conversations about lifestyle? • How do you motivate patients to change their lifestyle?*
Collaboration with primary care and social work services	*• What kind of referrals do you make concerning lifestyle counseling? • Can you tell me about your collaboration with primary care regarding to lifestyle counseling?• Can you tell me about your collaboration with social work services regarding lifestyle counseling?*
Continuation of collaboration with social work services	*• Can you tell me about your network of social work services?* *• When was the last time you referred a patient to social work services? • How do you refer a patient to social work services?*
Improvements for collaboration between general practices and social work services	*• What is working well in the collaboration with social work services regarding lifestyle counseling? • What would help to improve the collaboration with social work services regarding lifestyle counseling, in case this is necessary?*
General improvement points	*• Which improvements for providing lifestyle counseling have not yet been discussed?*

Before recordings were made, interviewees signed an informed consent that contained information about guarantee of anonymity and data management. The interviews were conducted in Dutch and took place between March 2024 and August 2024, physically at either the Radboud University Medical Center or the workplace of the participants. The individual interviews with GPs and PNs lasted approximately 45 min, the group interviews approximately 60 min. Both the individual and group interviews were conducted by 1 researcher. For the group interviews a second researcher was present. Recordings of the interviews were made using a voice recorder. Subsequently, the audio recordings were transferred to a secure data disk and transcribed.

### Data Analysis

To analyze the interviews, ATLAS.ti version 24.0.0 was used, allowing for both systematic coding and thematic analysis. For the systematic coding and thematic analysis, the approach described by Braun and Clarke (2006) was conducted.^31^ The analysis was inductive, focusing on identifying subthemes and themes that emerged from the data without imposing a predefined framework. One researcher coded and analyzed all the interviews, while another researcher independently coded and analyzed 2 individual and 2 group interviews to ensure consistency. The subthemes and themes were compared between the 2 researchers and discussed until consistency was reached. Some of the subthemes explicitly highlight areas that could be improved according to participants. These subthemes are discussed throughout the results.

Causal relationships within or between subthemes were noted by one of the coders during a review of the coded parts of the transcripts. Subthemes that included a causal relationship or that were causally related to other subthemes formed the basis for defining variables within the CLD.^32^ The other subthemes were not included. These can be found in Supplemental Appendix B. Subsequently, the full interview transcripts were carefully reviewed again by one of the coders to identify any overlooked perceived causal relationships within or between the subthemes. The overlooked relationships were added to the CLD. Explicitly mentioned relationships as well as implicitly mentioned relationships were included. See Table 3 for 2 examples of the analytical process. The CLD was discussed by the 2 coders until consensus was reached. Small iterations were made, such as merging some variables and changing variable names.

**Table 3.** Example of the Analytical Process From Raw Data to Subthemes, Themes, and CLD Variables.

Analysis steps	Example 1	Example 2	
Participant quote	*“I also hear from patients yes, good experiences back, so that does give me confidence to refer in the future as well.” – PN, P5*	*“I find the social domain very confusing. [. . .] I’m actually missing an overview about them” – PN, P8*	
Code(s)	Positive patient experience gives confidence to refer again	Overview social work services domain unclear	Overview social work services missing
Subtheme	Positive patient experiences	Need for an overview of social work services	
Theme	General practices referring to social work services	General practices’ overview and knowledge of social work services	
Variables and their relationship	Number of positive experiences of patients with social work services →+ GP/PNs’ trust in social work services	Degree to which GP/PNs have an overview of the roles of social work services →+ GP/PNs’ understanding of available social work services	
Implicit or explicit relationship	Explicit	Implicit	

The CLD was created using Vensim PLE version 10.2.0, as this software supports the visualization of complex systems. Within the CLD directional arrows are used to depict links between variables. Arrows with positive signs (+) denote that a change in variables occurs in the same direction (eg, an increase in variable A causes an increase in variable B, or a decrease in variable A causes a decrease in variable B). While negative signs (−) denote that a change occurs in the opposite direction (eg, an increase in variable A causes a decrease in variable B). Two types of feedback loops can emerge within the CLD: (a) *reinforcing* feedback loops amplify changes in the original variable, meaning a decrease leads to further reductions, while an increase drives further growth while (b) *balancing* feedback loops stabilize the system by counteracting changes. Colors were used in the CLD to visually indicate which variables belong to a certain main theme. Quotes were selected to illustrate relationships between variables, the quotes were translated from Dutch to English.

### Member Check

A group meeting was organized to validate the CLD, involving 2 GPs, 3 PNs, 1 community social worker, 1 health policy advisor, 1 advisor elderly care, and 1 coordinator of community support services who is also a wellbeing coach. Besides, separate member check meetings were held with 1 community sports coach and 1 coordinator of community support services who is also a wellbeing coach. To do so, again general practice owners and managers of social work services were asked which professionals could be contacted for participation. Most of the professionals who participated also took part in the interviews, except for the social worker, health policy advisor, advisor elderly care, and one of the coordinators of community support services. During the group meeting, participants were divided into 3 groups to ensure a diverse mix of professions in each group, while keeping the groups small enough for discussion. The CLD was divided into 4 sections of approximately equal size, in some cases by combining several main themes: (1) addressing lifestyle within general practices, (2) general practices referring to primary care, general practices referring to CLIs, (3) general practices’ overview and knowledge of social work services, and (4) general practices referring to social work services, contact between general practices and social work services, patient status exchange between GP/PNs and social work services. Each group or person was assigned to review at least 2 sections of the CLD. A semi-structured interview guide was followed, including questions like: What do you recognize? What do you not recognize? Is there missing information? Additionally, to stimulate discussion, participants were provided with 2 or 3 questions related to the themes. For instance, participants were asked: Can you provide an example of a situation in which a GP or PN and social work services interacted concerning a patient? Participants could give feedback either verbally or by making modifications to the sheet with the CLD. Consequently, all 4 sections underwent a double review. Recordings of the meetings were made using audio recorders, which were transferred to a secure data disk and transcribed. The member checks took place in October 2024 and interviewees signed an informed consent before participating.

The CLD was revised based on the feedback received. Newly mentioned variables were added to the CLD. There were no disagreements about the existing variables during the member check. Certain connections between variables were disagreed with and subsequently reviewed. When these connections were explicitly recognized by at least 1 person during the interviews or member check they were not removed, otherwise they were removed. This resulted in a final version of the CLD.

### Ethical Approval

As determined by the medical ethical review committee Oost-Nederland, ethical approval was not required for this study (file number: 2024-17126).

## Results

**Figure 1.**
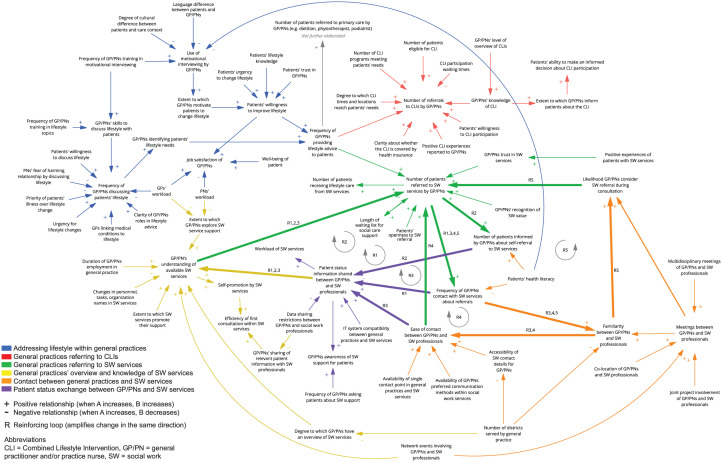
Causal loop diagram (CLD) about lifestyle counseling for chronic diseases within and between general practices and social work services, from the perspective of general practices.

Figure 1 presents the developed CLD. For each theme, both the variables and relationships within the CLD and the identified areas for improvement are discussed in the sections below. The last section describes the feedback loops within the CLD.

Links between variables indicate presumed causality, shown by directional arrows. Positive arrows (+) mean variables change in the same direction, while negative arrows (-) indicate opposite changes. Feedback loops are labeled as R1 to R5 and highlighted using bold lines. Two types of feedback loops can occur: (a) reinforcing loops amplify changes, driving variables to extremes, and (b) balancing loops counteract changes, promoting stability. The colors stand for the different themes, explained in the methods and results.

### Addressing Lifestyle Within General Practices (Top Left, Blue in CLD)

GPs and/or PNs (hereafter GP/PNs) see addressing lifestyle as an opportunity to assess patient’s lifestyle needs, which is a starting point for motivating them or giving lifestyle advice. The extent to which GP/PNs address lifestyle with patients is perceived to be influenced by various factors. These factors include among others GPs’ workload, PNs’ concerns that discussing lifestyle may strain the patient-provider relationship, and potential patient resistance. Concerns about patient-provider relationship is illustrated by the following quote:

But sometimes I find it [lifestyle conversation] quite challenging. Especially with patients who come in for annual check-ups and have chronic conditions—I really want to keep seeing them. I also want them to feel comfortable coming to me and not feel like, ‘Oh, here she goes again about smoking,’ to the point where I risk losing my patients. – PN, P5

Having the skills to discuss lifestyle effectively and to use motivational interviewing techniques can, according to GPs and PNs, enhance their ability to motivate patients. GP/PNs emphasize that regular training sessions are crucial for improving these skills. Perceived barriers for using motivational interviewing include language barriers and patients with low health literacy.

### General Practices Referring to CLIs (CLD: Top Right, Red)

The Combined Lifestyle Intervention (CLI) is mentioned as a referral option for lifestyle counseling for patients with obesity. In the Netherlands, CLI programs are 2-year lifestyle interventions for overweight or obese individuals, covered by health insurance since 2019. GP/PNs report misalignment of the CLI with patients’ needs as barrier, such as logistical challenges related to timing and location, as well as concerns about the program’s content (eg, the availability of a CLI program tailored for low literacy individuals). Additionally, participants noted that better knowledge of the CLI program is helpful to promote referrals, but also to inform patients more effectively about the program.

### General Practices Referring to Social Work Services (CLD: Center, Green)

Referral of patients to social work services is the center of the CLD. As illustrated in the CLD, numerous factors are perceived to impact the extent to which GP/PNs refer patients to social work services. Key factors include knowing what social work services have to offer, the ease of contacting the services, seeing opportunities for referral to the social work services during consultation, and having the trust that the social work services handle the healthcare questions appropriately. The latter is illustrated by this quote of a health policy advisor from the Municipality Heath Services:

Yes, you need to have trust in the person, in this case, the wellbeing coach in the ‘Wellbeing on Prescription’ program [social prescribing program]. You need to trust that the wellbeing coach will find something [. . .] that suits the patient and that your patient will truly benefit from it. – P12

GP/PNs state that trust in social work services grows with hearing positive experiences. A precondition for referring patients to social work services is the patient’s consent. Furthermore, waiting lists can increase as the number of referrals grows, and longer waiting lists may lead to fewer referrals to social work services.

### General Practices’ Overview and Knowledge of Social Work Services (CLD: Bottom Left, Yellow)

GP/PNs perceive determining whether a patient should receive help by social work services and choice of the appropriate organization for referral to be simpler if they have a better understanding of the roles and services provided. A comprehensive overview of what social work services have to offer can, according to GP/PNs, enhance their knowledge about the resources available. Additionally, networking events and keeping track of social work services (eg, checking their websites regularly) are recognized by GP/PNs to further enhance healthcare providers’ awareness of available resources.

Changes in personnel, roles, and organizational structures within the social work services makes provision of an overview more complex. Similarly, when GP/PNs are newly employed at a general practice, this could lead to them not being familiar with the resources available within social work services.

Moreover, when GP/PNs have more knowledge about the available resources, they recognize that they can better inform patients about these, as demonstrated by the following quote:

I might want more information about what options are available, you know, what the possibilities are. I think that would already be a big improvement. Then I might make more use of it. – GP, P6

### Contact Between General Practices and Social Work Services (CLD: Bottom Right, Orange)

As illustrated in the CLD, several strategies have been identified to improve mutual familiarity between GP/PNs and professionals from social work services. These include participation in multidisciplinary meetings, attending networking events, and sharing workspaces within the same building. Another strategy, emerging from the interviews, is the direct contact between GP/PNs with social work services in case patients themselves are unable to reach the social work services. GP/PNs and social work professionals recognize that mutual familiarity enhances GP/PNs’ awareness of social work services, which in turn increases the likelihood that these services are considered during patient consultations.

A community sports coach shared the following:

[. . .] I joined a network lunch last week. [. . .] I was sitting next to someone I’ve been working with [. . .]. And he said, ‘Yes, I know you are there, and I know how to find you and what you do, but I just don’t think about it enough.’ And through this conversation, he was reminded: oh yes, sports. So, I think it is really important to keep those lines short and stay visible with the different partners who are important. – P20

Due to overlapping neighborhoods in their working area of general practices, GP/PNs have to collaborate with several social services districts, and social service professionals have to collaborate with many GP practices. According to the interviewed GPs and PNs, this makes it challenging to become familiar with all social work professionals and their specific roles.

A GP reflects:

With this approach, people really need to have a GP in their own district; otherwise, it becomes unmanageable. – P9

For professionals, a single point of contact within each general practice or social work organization facilitates collaboration by simplifying communication. In addition, familiarity with other professionals and access to contact information further simplify communication.

### Patient Status Exchange Between GP/PNs and Social Work Services (CLD: Center, Purple)

According to the interviewees, a patient status report is occasionally provided to the GP/PN when the GP/PN directly refers patients to the social work services. However, when patients contact social work services on their own, a patient status report to the provider is unusual. When a patient status report is received, GP/PNs view it as enhancing their awareness of available resources within social work services, thereby facilitating future referrals. Privacy regulations and the use of separate IT- and organizational systems are mentioned in the interviews as barriers to establishing effective communication about patient status. While receiving patient status reports from social work services is 1 way for GP/PNs to monitor patient progress, another method is through active inquiry in follow-up consultations with patients.

### Feedback Loops in the CLD

Five reinforcing feedback loops were identified within the system (Figure 1, indicated as R1-R5). R1 represents a reinforcing feedback loop, which shows that when the GP/PN contacts social work services for a referral (eg, telephone interaction), this results in receiving more information about the patient’s status (eg, post-consultation update). Consequently, the GP/PN becomes more knowledgeable about social work services (eg, becoming more familiar with the responsibilities of community social workers), which is perceived to lead to more referrals to social work services. However, as illustrated by R2, the more patients that receive information to navigate social work services independently, the less patient status information is being shared by social work services with the GP/PN. As a result, the GP/PNs’ knowledge does not increase, nor do the number of referrals.

As illustrated by R3, when GP/PNs reach out to social work services, this encourages more frequent communication between the 2 parties. This enhanced communication is perceived to foster a better mutual familiarity (eg, the PN knows who is the community sports coach of the district), making it easier for professionals from social work services to contact the GP/PN and vice versa. This contributes to GPs/PNs receiving more patient status information from social work services, thereby increasing their knowledge of available services and leading to more referrals. R4 shows that when GP/PNs can easily contact social work services (eg, having their number in phone contacts), due to knowing each other, they perceive this as a factor that encourages more referrals to social work services. Finally, R5 illustrates that when GP/PNs are more familiar with the professionals from social work services, they believe this makes the social work services more present in their minds during consultations, which is regarded as contributing to an increase in referrals.

## Discussion

To the authors’ knowledge, this is the first study that maps the system of lifestyle counseling within and between general practices and social work services. The CLD illustrates the dynamics of intersectoral collaboration and provides valuable insights in options for improvement. To further enhance referrals to social work services, GP/PNs need a better understanding of the available referral options of social work services. The findings of this study suggest that GP/PNs gain a better understanding of social work services when they are provided with a comprehensive overview, engage in more network events with social work professionals, and exchange patient status information more frequently. Additionally, greater familiarity between GP/PNs and social work professionals can further support the referral process to social work services. This can be supported by more meetings between professionals and more frequent contact regarding referrals. Lastly, assigning a clear point of contact within both general practices and social work services could help streamline communication, which is a key factor in increasing referrals.

The CLD is a useful tool for exploring how areas for improvement can be addressed, as it highlights the variables that influence them. Among these are the so-called “leverage points,” which are variables that can be modified to induce desired changes elsewhere in the system. For example, one of the improvement options is that GP/PNs need to be more familiar with social work service resources. The CLD suggests several leverage points for achieving this, including creating comprehensive overviews of available services, organizing network events, promoting social work services, obtaining patient status information from social work services, and actively exploring these resources. Factors that negatively influence the familiarity of GP/PNs with social work services include changes in personnel, roles, and organizational names within social work services, as well as the relatively short duration of employment among GP/PNs in certain general practices. Both the leverage points and factors that can have a negative influence need to be considered when determining a strategy for improvement. However, the CLD does not give insight into which leverage points are most effective for enhancing the system of lifestyle counseling, since this depends on the context. It is recommended that professionals identify the barriers and areas for improvement within their own organization. By consulting a CLD relevant to their context, they can then identify the leverage points that contribute to improvement. From there, they can select those that are most suitable for their specific context.

Insights from this study’s CLD, or from versions adapted to other contexts can be applied by professionals in daily practice as well as by health policymakers. Certain leverage points can be directly integrated into professional routines. Health policymakers may include the CLD’s findings in their planning of intersectoral collaboration. Furthermore, professionals from general practices and social work services can use the various elements of the CLD to establish or further strengthen their collaboration plans and practice.

Several of this study’s findings correspond with literature. The CLD indicates that an increase in effective lifestyle conversations leads to more referrals of patients with a chronic disease from general practices to social work services. Although the literature does not state this relationship explicitly, it suggests that referrals to social work often originate from lifestyle counseling.^33^ Some previous studies have noted a lack of skills in lifestyle counseling among GP/PNs. While this study’s findings suggest that skills in facilitating effective lifestyle conversations are associated with more frequent discussions about lifestyle, the interviewees themselves did not report a lack of skills.^15-17^

Besides, it was observed that the level of trust that GP/PNs have in social work services is directly correlated with their propensity to refer patients to them. This trust can be modulated by their experiences with prior referrals, which can be either positive or negative. Previous research also identified this trust as an important determinant in the referral process.^19,22,34^ Furthermore, the findings indicate that patients’ receptiveness to being referred to social work services plays a critical role. Previous studies indicate that a patient’s motivation and willingness may act as a barrier for a referral.^19,22,34^ Also, the unfamiliarity of GP/PNs with social work services observed in this study aligns with existing literature, indicating that GP/PNs often lack awareness of the available social work services and how to access them.^22,24^ The findings of this study suggest that a clear overview of social work support and participation in network events could help increase referrals from general practices to social work services. Both findings are supported by previous studies.^19,22,34^ In earlier studies, the lack of proven effectiveness of interventions was also described as a barrier to referring.^14,35^ However, the interviewees did not express doubts about this.

This study found that communication between GP/PNs and social work services could be improved by assigning a clear point of contact within each general practice and social work organization. Social prescribing programs in the Netherlands and the United Kingdom even take it a step further by using a single designated contact point through which general practices can refer patients to social work services.^21,34,36^ Considering both findings, it remains unclear whether it is preferred to have a single point of contact for the entire social domain or 1 point of contact for each social work organization.

Finally, it was found that privacy regulations and fragmented IT systems hinders the exchange of information about a patient’s situation between general practices and social work services. While, as literature on the medical domain indicates, access to relevant information from other healthcare professionals is essential for enhancing patient safety and quality of care.^37,38^

A limitation of this study is the potential for socially desirable responses. Although participants were informed that their answers would remain anonymous, the interview setting may still have influenced them to respond in a socially desirable manner. A limited number of professionals were interviewed. However, the intention was not to reach data saturation for each profession interviewed. The intention was to capture the views of a diverse group of professionals, including GPs, PNs, and 5 different professions from social work organizations. It would be valuable in future research to include additional interviews to obtain an even more comprehensive overview of the variables and relationships between the variables.

Another limitation of this study is that the CLD was based on qualitative data from a few districts in Nijmegen, a city in the Netherlands. Because social work services in the Netherlands are typically organized at the municipal level their structure and collaboration vary considerably across locations. The results of the CLD are therefore less representative of other locations in the Netherlands or of international contexts. It is advised to use the CLD as a starting point and further adapt it to other geographical and organizational contexts. It would be specifically interesting to explore the CLD in rural areas, where collaboration between general practices and social care services may differ.

Besides, the focus of the current CLD is on collaboration of GP/PNs and social work services. Professionals involved in CLI programs were not involved, nor were physiotherapists and dieticians. Nevertheless, given the relatively large amount of information received about the CLI from GPs and PNs, this was incorporated into the analysis. Future studies could include other (care) professionals to gain a more comprehensive understanding of how the CLI and their primary care services are integrated into the lifestyle counseling system. The patient perspective was also beyond the scope of this study, although incorporating it in future research could enrich the CLD. It might lead to the addition of factors that influence patients’ engagement with lifestyle counseling in general practice and social work settings, as well as their perspectives on collaboration between these domains.

Furthermore, the CLD contains only a limited number of feedback loops. This suggests that the system either contains relatively few feedback loops or that additional variables or connections may not have been identified. The overall system is likely more complex and extensive than the mapped version. The model represents only the aspects that emerged from the interviews, providing a partial but meaningful view on the broader system of lifestyle counseling within and between primary care and social work services.

It can be considered a strength that conversations were held with professionals from both general practices and social work services, as this captures perspectives from both sides of the collaboration. During the member-check process, these professionals engaged in discussions about the CLD, allowing them to ask questions and clarify specific relationships within the system. This makes the final CLD a reliable representation of how the involved professionals perceive the system.

## Conclusion

To provide effective lifestyle counseling for patients with chronic diseases in general practice, optimal collaboration with non-medical professionals from social work services is essential. The interviews highlighted areas where collaboration could be improved, while the CLD illustrates how these areas for improvement are interrelated and highlights potential leverage points for addressing them. Based on the CLD and interviews, recommended actions include developing a clear overview of available social work services and their roles for GPs and PNs. In addition, opportunities should be created for GPs, PNs, and social service professionals to become more familiar with each other and each other’s work, for example through joint meetings. Furthermore, more frequent and structured communication between general practices and social work services should be established.

## Supplemental Material

sj-docx-1-jpc-10.1177_21501319251412648 – Supplemental material for Dynamics of Lifestyle Counseling for Chronic Diseases Within and Between General Practices and Social Work Services Causal Loop Diagram and Points for Improvement Supplemental material, sj-docx-1-jpc-10.1177_21501319251412648 for Dynamics of Lifestyle Counseling for Chronic Diseases Within and Between General Practices and Social Work Services Causal Loop Diagram and Points for Improvement by Demi E. van Os, Bart H. L. Ament, Suzanne A. Ligthart, Gerdine A. J. Fransen and Willem J. J. Assendelft in Journal of Primary Care & Community Health
