# Supplementary material for: Dynamics of Lifestyle Counseling for Chronic Diseases Within and Between General Practices and Social Work Services Causal Loop Diagram and Points for Improvement
Source: J Prim Care Community Health. 2026 Jan 16;17:21501319251412648. doi: 10.1177/21501319251412648 (PMC12812193; doi:10.1177/21501319251412648)
Supplement: sj-docx-1-jpc-10.1177_21501319251412648 – Supplemental material for Dynamics of Lifestyle Counseling for Chronic Diseases Within and Between General Practices and Social Work Services Causal Loop Diagram and Points for Improvement [file sj-docx-1-jpc-10.1177_21501319251412648.docx]

**APPENDIX A. Interview guide for group interviews**

Table A1. Topics and examples of probing questions outlined in the interview guide for group interviews with social work services.

| **Topics regarding lifestyle counseling** | **Examples of probing questions** |
| --- | --- |
| Role | *• What is your role regarding lifestyle counseling? • What do you offer resident in the field of lifestyle counseling? • Which groups of residents do you focus on?* |
| Collaboration with general practices and social work services | *• Which organizations do you work with when it comes to lifestyle counseling? • How do residents find you? • When do you refer residents to another healthcare professional?* |
| Continuation of collaboration with general practices | *• In which way do you have contact with general practices? • When do general practices refer to social work services? • When was the last time you saw a resident who was referred from a general practice? • How do costs and reimbursements play a role in referral to social work services?* |
| Improvements for collaboration between general practices and social work services | *• What is working well in the collaboration with general practices regarding lifestyle counseling? • What would help to improve the collaboration with general practices regarding lifestyle counseling, in case this is necessary?* |
| General improvement points | *• Which improvements for providing lifestyle counseling have not yet been discussed?* |

**APPENDIX B. Subthemes not incorporated in the causal loop diagram**

- Lifestyle improvements lead to better health outcomes
- Providing lifestyle advice is often the role of the practice nurse
- Patients are key actors in managing their lifestyle
- Lifestyle change should be done in small steps
- Trust between patient and caregiver is important in lifestyle conversations
- Financial considerations of patients can affect referral decisions
- Value of one central contact for general practices covering the entire social domain
- Integrating the social domain in computer system of general practices can be beneficial
- Type and severity of health-related problems differ between neighborhoods
- General practices and social work services speak differently about lifestyle
- General practices and social work services differ in their perspectives on lifestyle
- The potential of social work services to reduce workload in general practices
